# An hour-specific transcutaneous bilirubin nomogram for Mongolian neonates

**DOI:** 10.1007/s00431-015-2536-2

**Published:** 2015-04-14

**Authors:** Moe Akahira-Azuma, Naohiro Yonemoto, Rintaro Mori, Shinichi Hosokawa, Takeji Matsushita, Khulan Sukhbat, Gerelmaa Nansal, Bayasgalantai Bavuusuren, Enkhtur Shonkhuuz

**Affiliations:** Department of Pediatrics, National Center for Global Health and Medicine, 1-21-1 Toyama, Shinjuku-ku, Tokyo, 162-8655 Japan; Department of Neuropsychopharmacology, National Institute of Mental Health, National Center of Neurology and Psychiatry, 4-1-1 Ogawahigashimachi, Kodaira, 187-8553 Tokyo Japan; Department of Health Policy, National Center for Child Health and Development, 2-10-1 Okura, Setagaya-ku, 157-8535 Tokyo Japan; National Center for Maternal and Child Health of Mongolia, Huvisgalchid Street, Bayangol District, Ulaanbaatar, 16060 Mongolia

**Keywords:** Neonatal hyperbilirubinemia, Transcutaneous bilirubin, Hour-specific nomogram, Mongolian

## Abstract

Transcutaneous bilirubin (TcB) nomograms have been developed for different populations. However, the TcB level, rate of rise and peak varies among countries and ethnicities. The aim of this study was to establish an hour-specific TcB nomogram for healthy term and late preterm Mongolian neonates during the first 144 h after birth. A total of 5084 TcB measurements from 1297 healthy neonates (gestational age ≥35 weeks, birth weight ≥2000 g) were obtained from October 2012 to October 2013. All measurements were performed using the Jaundice Meter, the JM-103 at 6 to 144 postnatal hours. Mongolian infants had the following characteristics: 27.1 % were delivered by cesarean section, 17.8 % had a birth weight >4000 g, and >90 % were being breastfed. TcB percentiles for each designated time point were calculated for the development of an hour-specific nomogram. TcB levels increased most rapidly in the first 24 h and less rapidly from 24 to 78 h, reaching a plateau after 78 h for the 50th percentile. TcB levels of Mongolian neonates for each time point were higher than those of previous studies.

*Conclusion*: The higher values of the TcB nomogram for Mongolian neonates may be due to their Asian ethnicity and exclusive breastfeeding.

**Table:** 

**What is Known:** *• TcB nomograms for neonatal jaundice screening have been established for many countries and ethnicities. The pattern of the TcB nomogram varies by country and ethnicity.*
**What is New:** *• A TcB nomogram for neonates of Mongolian ethnicity at 6–144 postnatal hours was created and it had higher values than those in previous studies.*

## Introduction

Neonatal hyperbilirubinemia and neurological sequelae resulting from kernicterus pose a serious medical burden in both developing and developed countries [13, 15, 16, 23]. However, severe hyperbilirubinemia in the newborn is preventable through appropriate follow-up, diagnosis, and treatment, such as phototherapy and exchange transfusions [8, 9, 13, 23]. The American Academy of Pediatrics (AAP) recommends that all neonates undergo total serum bilirubin (TSB) or transcutaneous bilirubin (TcB) measurements at least once before hospital discharge to assess their risk of hyperbilirubinemia [2, 17].

TcB nomogram is a useful tool for neonatal hyperbilirubinemia screening [10]. However, the TcB value, rate of rise and peak varies with country and ethnicity [5], suggesting that each country and ethnic group has specific risk factors for hyperbilirubinemia. Few studies on the TcB nomogram have been done for Mongolian neonates in a resource-limiting setting [1].

The aim of this study was to create an hour-specific TcB nomogram for healthy Mongolian neonates, which was achieved by obtaining TcB measurements in the first 144 h after birth.

## Methods

### Patients

This was a prospective cohort study at the National Center for Maternal and Child Health (NCMCH), a tertiary care hospital in Ulaanbaatar, Mongolia. Neonates who were eligible for enrollment were healthy newborns ≥35 weeks of gestation with a birth weight ≥2000 g who were delivered in the hospital on a weekday (from Monday to Friday morning) and whose address were in the Bayangol district. Exclusion criteria included the following: infants with birth asphyxia, respiratory distress, NICU admission, congenital infection, Rh isoimmunization, jaundice within 24 h of birth, skin infection, congenital spinal anomaly, those with no TcB measurement, and cases with missing information. The study was carried out from October 15, 2012 to October 4, 2013 but was temporarily suspended between June 28 and August 2, 2013 when the maternity unit was closed for renovation. Perinatal information on the mother and infant were extracted from their medical charts including sex, gestational age, birth weight, mode of delivery, duration of hospitalization, maternal age, parity, gravidity, maternal blood type, family history of siblings, and feeding type before and after discharge.

This study was approved by the NCMCH ethics committee in Mongolia and the National Center for Global Health and Medicine (NCGM) human investigation ethics committee in Japan, in accordance with the Declaration of Helsinki. Informed consent was obtained from all individual participants (mothers of the infants) included in the study.

### Measurements

TcB measurements were performed between 9 am and 5 pm twice daily (once in the morning and once in evening) from Monday to Friday. The Jaundice Meter JM-103 (Konica Minolta, Osaka, Japan) was used by properly trained pediatricians or a research technician to measure TcB. The operation of the JM-103 and its measurement technique have been described previously [1]. Briefly, TcB levels were measured at the forehead and at the midsternum three times at each site, and the median value was defined as the TcB at each measurement site. The highest value of two sites was used as the TcB for each infant. Because NCMCH applies a discharge policy of ≥48 h for vaginally delivered infants and ≥72 h for those delivered by cesarean section, the parents were advised to return to the outpatient clinic for follow-up measurements. Enrolled neonates returned for a follow-up visit between days 3–6 or earlier if the parents noticed worsening jaundice in their child. Parents who did not bring their child for the visit were reminded by telephone. An enrollment number was given to each neonate and recorded in a log book to identify the study neonate during the visit. TcB values were recorded on a flow sheet designed for the study and attached to the signed informed consent form in the medical record of each infant.

TcB measurements obtained at 6 ± 2 h intervals up to 144 h were examined [12, 18]. TcB measurements not taken between these time measurements were omitted from analysis. The decision regarding the need for phototherapy was made by the attending pediatrician based on TSB levels, in accordance with NCMCH’s protocol and/or AAP guidelines [2]. If TcB values were obtained, measurement values obtained after the initiation of phototherapy were excluded from analysis.

The characteristics of mothers and children are presented as percentages for binary and categorical data, and as means and standard deviations for continuous data. The data analysis was performed by using SPSS 15.0 for Windows (SPSS, Chicago, IL).

TcB percentiles (95th, 90th, 75th, 50th, 25th, 10th, and 5th percentiles) were calculated for each designated time. A TcB nomogram with smoothed percentile curves for the period 6–144 postnatal hours was prepared using Microsoft Excel 2010 (Microsoft, Redmond, WA).

## Results

**Fig. 1:**
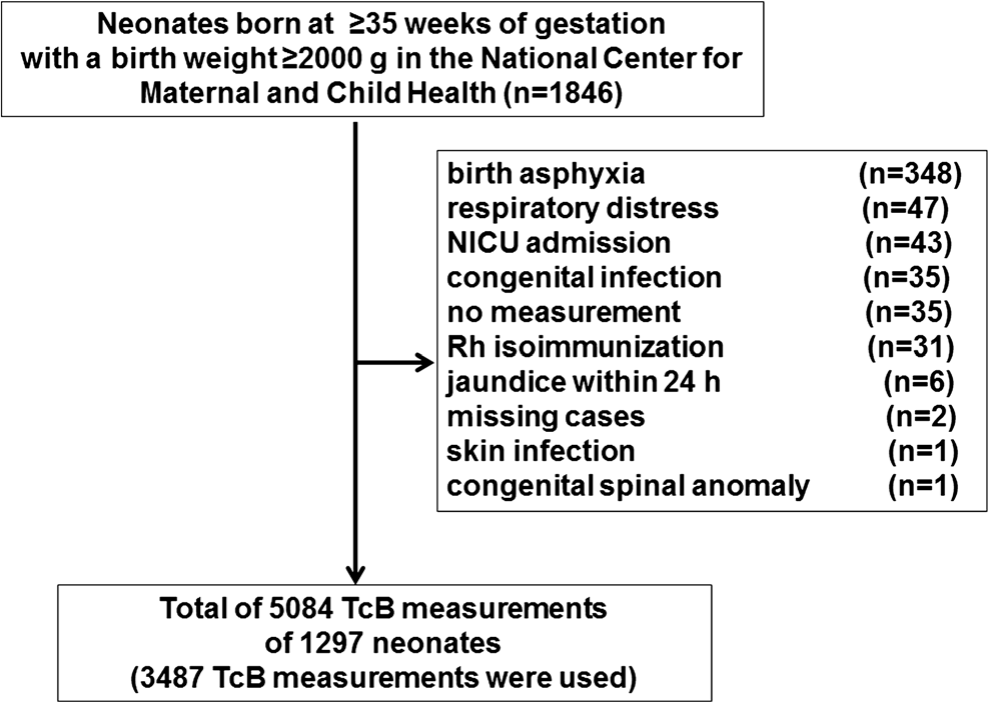
Study flow

Of 1846 live births in NCMCH during the study period, 549 neonates did not meet the enrollment criteria (Fig. 1), resulting in 1297 healthy term and late-preterm neonates from whom 5084 TcB measurements were obtained. Among these TcB measurements, 3487 that were taken at the designated time were used for analysis. Of the 1297 enrolled neonates, 1137 (87.7 %) returned for the follow-up visit after hospital discharge.

Demographic and background data of mothers and neonates are summarized in Table 1. Of note, all neonates were Mongolian, 27.1 % were delivered by a cesarean section, ≥90 % were breastfed, and 17.8 % had a birth weight ≥4000 g. Regarding the family history of jaundice, 851 of 1297 enrolled infants had siblings and 163 of 851 siblings (19.2 %) had a history of treatment for jaundice according to an interview with the mother. Details of the jaundice could not be obtained to clarify the etiology of jaundice.

**Table 1:** Characteristics of the enrolled neonates and their mothers

Characteristic		*n* = 1297	(%)
1. Sex	Male	662	(51.0)
	Female	635	(49.0)
2. Gestational age	35–36 weeks	16	(1.2)
	37–40 week	1230	(94.9)
	41–42 weeks	51	(3.9)
3. Birth weight	2000–2999 g	92	(7.1)
	3000–3999 g	975	(75.1)
	4000–4999 g	228	(17.6)
	>5000 g	2	(0.2)
4. Mode of delivery	Vaginal	945	(72.9)
	Cesarean section	352	(27.1)
5. Duration of hospitalization	1–2 day (s)	907	(69.9)
	3–4 days	355	(27.4)
	5–7 days	35	(2.7)
6. Maternal age	15–24 years old	293	(22.6)
	25–34 years old	748	(57.7)
	35–45 years old	256	(19.7)
7. Parity	1	320	(24.7)
	2	411	(31.7)
	3	258	(19.9)
	4	169	(13.0)
	5 to 9	139	(10.7)
8. Gravidity	1	451	(34.8)
	2	541	(41.7)
	3	230	(17.7)
	4	60	(4.6)
	5 to 9	15	(1.2)
9. Maternal blood type	O	366	(28.2)
	A	347	(26.8)
	B	456	(35.2)
	AB	116	(8.9)
	Unknown	11	(0.8)
	No description	1	(0.1)
10. Family history of siblings	First baby	446	(34.4)
	Phototherapy	161	(12.4)
	Exchange transfusion	2	(0.2)
	No jaundice	688	(53.0)
11. Feeding (before discharge)	Breastfeeding	1264	(97.4)
	Formula	3	(0.2)
	Mixed	30	(2.3)
12. Feeding (follow-up visit)	No follow-up visit	160	
	Attended follow-up visit	1137	
	Breastfeeding	1025	(90.1)
	Formula	1	(0.1)
	Mixed	111	(9.8)

**Fig. 2:**
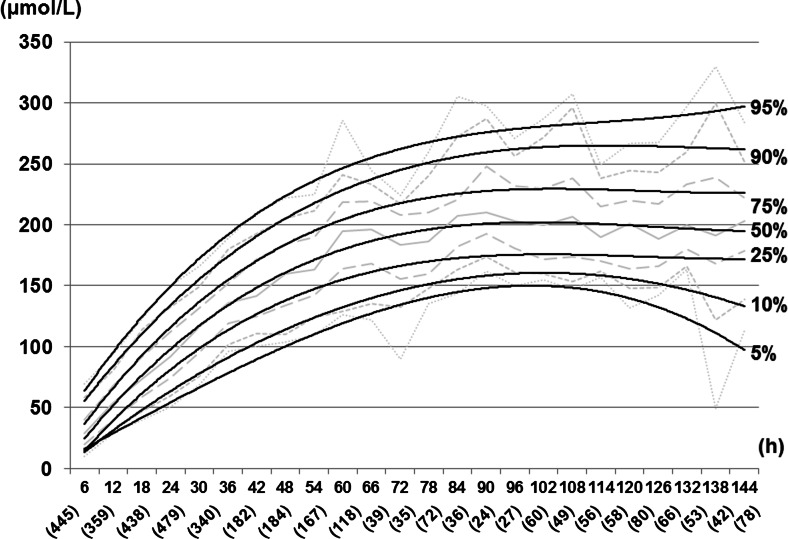
An hour-specific TcB nomogram for Mongolian neonates with smoothed curves for the 5th, 10th, 25th, 50th, 75th, 90th, and 95th percentiles. *Numbers in parentheses* indicate the number of TcB measurements at each time point

A TcB nomogram with smoothed percentile curves (95th, 90th, 75th, 50th, 25th, 10th, and 5th percentiles) for the time period 6–144 postnatal hours is presented in Fig. 2. The numbers of TcB measurements are shown for each time point. TcB levels increased in a linear fashion most rapidly in the first 24 h and less rapidly from 24 to 78 h, reaching a plateau after 78 h for the 50th percentile.

## Discussion

We created an hour-specific TcB nomogram for the first 144 h after birth for Mongolian healthy term and late preterm neonates. The characteristics of this group of Mongolian neonates included delivery by cesarean section in 26.9 % of cases and exclusive breastfeeding in >90 %. The TcB values and rate of rise at each designated time point resulted in a TcB nomogram with higher values than those reported for other countries and ethnicities in previous studies. This result indicates that this group might have risk factors and a higher likelihood of developing neonatal hyperbilirubinemia.

Many TcB nomogram studies have been performed in North America, Europe, and Asia in different groups [3, 6, 7, 11, 12, 14, 18, 19, 21, 25, 28] since the second generation of the TcB meter became available. The risk factors of hyperbilirubinemia were determined to be Asian ethnicity, exclusive breastfeeding, weight loss, and a sibling with neonatal jaundice [17]. In a North American study investigating an hour-specific TcB nomogram in the first 96 h using the JM-103 to measure levels in 3984 neonates, 73.1 % were Caucasian, 45.1 % were delivered by cesarean section, and 66.2 % were breastfed [18]. TcB nomogram for Mongolian neonates showed much higher values than the North American nomogram at each time point. This might be due to the predominant Caucasian ethnicity of the study group [22]. In a study on infants of Asian ethnicity, a TcB nomogram was developed using JM-103 measurements taken from 6035 Chinese infants [28]. These Chinese infants were delivered by cesarean section in 55.5 % of cases, 25.1 % were breastfed, and 9.4 % had a birth weight >4000 g. In contrast, our study of Mongolian neonates showed higher TcB values at each time point, suggesting that exclusive breastfeeding might have contributed to this result [4]. A recent study on the influence of dehydration on body weight loss at 72 h after birth in Taiwan revealed the optimum body weight loss cutoff points for predicting hyperbilirubinemia [27]. Unfortunately, the body weights on each day of TcB measurement were not determined in our study. Therefore, it remains unclear whether the acceleration of hyperbilirubinemia of Mongolian neonates is caused by exclusive breastfeeding itself or by dehydration secondary to inappropriate breastfeeding.

This prospective cohort study was a tertiary hospital-based study that enrolled neonates from one district, and TcB measurements were done either in the maternity unit or in the outpatient clinic [26]. Our study method for creating a TcB nomogram is practical and can be applied in countries with limited resources. This encourages resource-limited countries to create country- and ethnic-specific TcB nomograms.

This study has several limitations. First, there were insufficient numbers of TcB measurements at the follow-up visit on day 3–6, only one to two measurements. Second, the designated times for TcB measurements were difficult to establish, leading to the omission of a certain number of TcB measurements. However, it was impossible to fix a designated TcB measurement time for each neonate in ordinary practice. Third, the glucose-6-phosphate dehydrogenase (G6PD) and direct Coombs test are not routinely performed in prenatal care screening in Mongolia so G6PD deficiency and ABO incompatibility could not be ruled out in this group. Fourth, cephalohematoma, a risk factor for hyperbilirubinemia, might be increased, considering the high percentage of vaginal deliveries and heavy infants (17.8 % had birth weight >4000 g) [20, 24]. Although cephalohematoma should have been identified as a risk factor, this does not affect the validity of the nomogram. Fifth, because infants with significant hyperbilirubinemia were referred to the hospital for further work-up and treatment and were later excluded from the study, the TcB measurements and curves we report here might be lower than the actual TcB values.

The findings in this study contribute to improving appropriate diagnosis and treatment of neonatal hyperbilirubinemia, and might reduce the incidence of neurological impairment in this high-risk ethnic group.

## Conclusion

We provide data on TcB levels for the first 144 postnatal hours that were derived from a group of Mongolian term and late preterm neonates. The higher values of the TcB nomogram derived from Mongolian neonates may be due to their Asian ethnicity and exclusive breastfeeding.

## Abbreviations

AAP: American Academy of Pediatrics
G6PD: Glucose-6 pyruvate deficiency
GA: Gestational age
TcB: Transcutaneous bilirubin
TSB: Total serum bilirubin
